# Nutritional Strategies to Improve Meat Quality and Composition in the Challenging Conditions of Broiler Production: A Review

**DOI:** 10.3390/ani13081386

**Published:** 2023-04-18

**Authors:** Janghan Choi, Byungwhi Kong, Brian C. Bowker, Hong Zhuang, Woo Kyun Kim

**Affiliations:** 1 US National Poultry Research Center, USDA-ARS, Athens, GA 30605, USA; choij@uga.edu (J.C.); byungwhi.kong@usda.gov (B.K.);; 2 Department of Poultry Science, University of Georgia, Athens, GA 30602, USA

**Keywords:** broilers, meat quality, meat composition, feed composition, bioactive compounds, plant polyphenol compounds

## Abstract

**Simple Summary:**

Intense genetic selection and improvements in nutrient programs have enhanced the growth performance, feed efficiency, and meat yield of broiler chickens. The quality characteristics and nutrient composition of poultry meat are also crucial factors in poultry production. Many challenging conditions, including fast growth, pathogen infection, high rearing temperature, and feed contamination, can negatively influence poultry meat quality and composition. Studies have demonstrated that modulating the nutrient composition of feed and supplementing bioactive compounds, including vitamins, probiotics, prebiotics, exogenous enzymes, polyphenol compounds, and organic acids, have positively influenced the meat quality and body composition of broiler chickens.

**Abstract:**

Poultry meat is becoming one of the most important animal protein sources for human beings in terms of health benefits, cost, and production efficiency. Effective genetic selection and nutritional programs have dramatically increased meat yield and broiler production efficiency. However, modern practices in broiler production result in unfavorable meat quality and body composition due to a diverse range of challenging conditions, including bacterial and parasitic infection, heat stress, and the consumption of mycotoxin and oxidized oils. Numerous studies have demonstrated that appropriate nutritional interventions have improved the meat quality and body composition of broiler chickens. Modulating nutritional composition [e.g., energy and crude protein (CP) levels] and amino acids (AA) levels has altered the meat quality and body composition of broiler chickens. The supplementation of bioactive compounds, such as vitamins, probiotics, prebiotics, exogenous enzymes, plant polyphenol compounds, and organic acids, has improved meat quality and changed the body composition of broiler chickens.

## 1. Introduction

Poultry meat is one of the most inexpensive and popular protein sources in the world [1]. Furthermore, poultry meat is known to have a low fat content and high concentrations of omega-3 fatty acids, which can be beneficial for humans’ vascular health [2]. In 2019, the consumption rates of poultry meat, per capita in the world and the US, were approximately 14.7 kg (OECD-FAO) [3], which were higher than those for any other meat sources, including pork, beef, sheep, and goat meat [4]. It is expected that worldwide poultry meat consumption will continue to increase annually due to an increase in the global population and preference [5]. To meet this growing demand for poultry meat, there have been significant enhancements in broiler meat production. For instance, while 42-day-old broilers weighed around 586 g with a feed conversion ratio of 2.8 in 1957 [6], modern broilers weigh around 3278 g with a feed conversion ratio of 1.55 at 42-days-old, according to Cobb500 broiler performance and nutrition supplement 2022 [7]. Broiler meat is considered the most cost-effective and sustainable animal protein source because of the birds’ efficiency in converting feed to meat [8,9].

While effective genetic selection and nutrition programs have dramatically improved the productivity and efficiency of the broiler industry, fast-growing broilers more frequently show unfavorable meat quality compared to slow-growing broilers [10]. Poultry production continues to face a diverse range of challenges, including feed cost issues, bacterial and parasitic infection, heat stress, mycotoxins, etc., which can result in altered body composition, increased food safety concerns, and reduced meat yield and quality [11]. Negatively affected meat quality parameters, including water holding capacity (WHC), myopathies, texture, and flavor can influence consumer acceptance [12]. Nutrition is known to have a substantial influence on the meat quality and body composition of broilers [13]. This review summarizes (1) the effects of the diverse, challenging conditions in modern broiler production on meat quality and body composition and (2) the effects of nutritional strategies on the meat quality and body composition of broilers.

## 2. Challenging Conditions in Broiler Production Are Associated with Meat Quality

### 2.1. Fast Growth Rate

Through more than 70 years of intense genetic selection and effective nutritional programs, increased body weight, growth rates, and feed efficiency have been achieved among modern broiler chickens [14]. Genetic selection programs and nutritional programs have been designed to produce broilers with higher breast meat yields and body weight without considering fat accumulation, resulting in high levels of fat accumulation in modern broilers [15,16,17,18]. The increased production of breast meat has resulted in a decrease in leg meat production in modern broilers due to a change in body shape [19]. Modern broilers contain approximately 15 to 20% fat, and the majority (>85%) of the fat is not necessary for broilers’ body function [20]. In fast growth, the imbalanced ratio between oxidants and antioxidants in the body and the limited oxygen supply compared to the demand required for fast growth can lead to oxidative stress and hypoxia, respectively, resulting in lipid peroxidation and woody breast (WB) in modern broiler chickens [21,22,23,24]. Differences in meat quality have been observed between slow- and fast-growing broilers, as shown in Table 1. Overall, fast-growing broilers showed lower protein and higher fat contents compared to the slow-growing broilers. However, the differences in meat quality parameters, such as the pH, color, and WHC, in slow- and fast-growing broilers were not consistent—potentially because the slow-growing broiler strains varied across studies. According to Che et al. [25], the incidence rates of spaghetti meat (SM), severe WB, and mild white striping (WS) were nearly 36, 12, and 96%, respectively, and approximately 85% of breast meat showed several myopathies in Ontario, Canada. While there were no surveys that compared the incidence of breast myopathies between fast-growing and slow-growing broilers, the prevalence of breast myopathies would be greater in fast-growing modern broilers, which may be due to higher breast meat yield and higher oxidative stress compared to slow-growing broilers [26]. In addition, Zhang et al. [27] and Popova et al. [28] suggested that fast-growing broilers, which are slaughtered at a young age (D 42), may contain lower polyunsaturated fatty acids compared to the slow-growing chickens (raised past D 42) because older chickens tend to have higher polyunsaturated fatty acid contents in their breast meat. Hence, whereas modern broilers have higher yields of meat compared to slow-growing broilers, the meat of modern broilers may have higher fat content, lower omega-3 polyunsaturated fatty acid proportions, and more muscle myopathies.

### 2.2. Bacterial Infection

Bacterial infections, which can be caused by unfavorable rearing conditions and high stocking density, would be critical in broiler production [33]. The common pathogenic and infectious bacteria in broiler production include *Staphylococcus aureus*, *Campylobacter jejuni*, *Salmonella* spp., *Clostridium perfringens*, *Escherichia coli*, etc. [34]. Those bacterial infections can negatively affect the meat production and quality of broiler chickens, potentially via decreasing nutrient utilization, inducing inflammation, and influencing food safety [35,36,37]. For example, invasive food-borne pathogens, including *Salmonella* spp., *C. jejuni*, and *S. aureus* have been detected in chicken meat [38,39,40]. A previous study by Wang et al. [41] demonstrated that *Salmonella* Enteritidis infection increased fat accumulation by increasing lipid synthesis and reducing lipid transportation in the liver, which can potentially affect fat accumulation within the bodies of the chickens. Sadeghi et al. [42] demonstrated that *S.* Enteritidis infection increased the concentration of plasma malondialdehyde (MDA), an indicator for lipid peroxidation, and reduced the plasma total antioxidant capacity, thus potentially resulting in increased lipid peroxidation in the chicken meat. Likewise, many studies have shown that *Campylobacter* infection can induce systemic oxidative stress, which has the potential to induce lipid peroxidation in muscle [43,44,45]. However, more studies are needed to investigate the interactions between bacterial infection during the pre-harvest period and the meat quality of broiler chickens.

### 2.3. Coccidiosis

Coccidiosis is an enteric disease caused by *Eimeria* spp. in chickens, and it is a prevalent disease all over the world [46]. Cornell et al. [47] reported that approximately 95% of flocks in the western US using antibiotic-free production were infected with *Eimeria* spp. The seven species of *Eimeria*, including *Eimeria acervulina*, *E. maxima*, *E. tenella*, *E. brunetti*, *E. necatrix*, *E. mitis*, and *E. praecox*, colonize different regions of the gastrointestinal tract [48]. The most common *Eimeria* spp. found in chickens include *E. tenella* (ceca), *E. necatrix* (ileum), *E. acervulina* (duodenum), and *E. maxima* (jejunum) [49]. Many studies have reported that infection with *E. acervulina* and *E. maxima* reduced nutrient digestibility, especially amino acid (AA) digestibility, in broiler chickens [50,51]. Reduced AA digestibility may lead to decreased meat yield because AAs are building blocks for proteins in meat [52]. Our previous study demonstrated that *E. tenella* infection reduced the production of cecal short-chain fatty acids (SCFA; an important energy source for the host), which can lead to energy imbalances and reduce fat accumulation, resulting in decreased meat yields among broiler chickens [53,54]. Induced inflammation and oxidative stress caused by *Eimeria* infection can negatively affect meat quality in broilers [55,56]. Moreover, increased gut permeability caused by *Eimeria* infection can aggravate the negative effects of bacterial infection on meat safety and quality in broiler chickens [57]. Several studies have demonstrated the effects of *Eimeria* infection on meat quality in broiler chickens (Table 2). *Eimeria* infection induced lipid peroxidation in the breast meat, according to Cha et al. [58] and Partovi et al. [59]. Though conducted under identical experimental conditions, Qaid et al. [60] reported *E. tenella* infection increased lightness, while Qaid et al. [61] showed that *E. tenella* infection decreased lightness in the breast meat. Nonetheless, reduced growth performance due to *Eimeria* infection could both negatively and positively affect meat quality, because the fast growth rate of modern broilers can negatively influence meat quality and *Eimeria* infection may slow the growth rate.

### 2.4. Heat Stress

Heat stress, referred to as an imbalance between body heat production and loss, induces metabolic disruption and oxidative stress, which leads to physiological and behavioral alterations in broiler chickens [62]. Global warming is expected to accelerate the heat stress issues in broiler production [63]. Heat-stressed chickens have impaired growth performance along with poor meat quality, resulting in lower production efficiency and consumer acceptance [64]. The impact of heat stress on broiler meat quality is summarized in Table 3. According to Tavaniello et al. [65], heat stress reduced protein content and increased fat content in broiler chicken breast meat. Zhang et al. [66] and Zaboli et al. [64] demonstrated that heat stress increased the secretion of corticosterone, which is a critical stress hormone, reducing protein accumulation via protein degradation and increasing fat accumulation potentially via modulating the expression of fatty acid transport proteins and insulin receptors in broiler chickens. Heat stress increased the pH of breast meat, according to some studies [67,68]. It is hypothesized that heat stress induces the exhaustion of muscle glycogen antemortem and reduces postmortem glycolysis, which results in increased meat pH levels [69]. There were inconsistent results regarding the color of breast meat in heat-stressed broilers, as shown in Table 3. The potential reasons would be (1) increased pH due to the depletion of glycogen in the heat stress conditions resulting in a darker color of meat [70]; and (2) heat stress can denature sarcoplasmic proteins, which leads to the scattering of light, and this increases the lightness of meat [66]. However, a study by Zhang et al. [66] reported that heat stress could induce pale, soft, exudative (PSE)-like breast meat (e.g., low pH levels and pale coloring) in broilers. The differences might originate from different experimental conditions, such as diets, broiler strains, and management. 

### 2.5. Mycotoxins

Mycotoxins, the secondary metabolites of fungi, such as the genera *Fusarium*, *Aspergillus*, etc., are frequently found in poultry feed [72,73]. There are more than 400 mycotoxins with different absorption rates and effects on broiler chickens, as summarized in Table 4.

**Figure 1:**
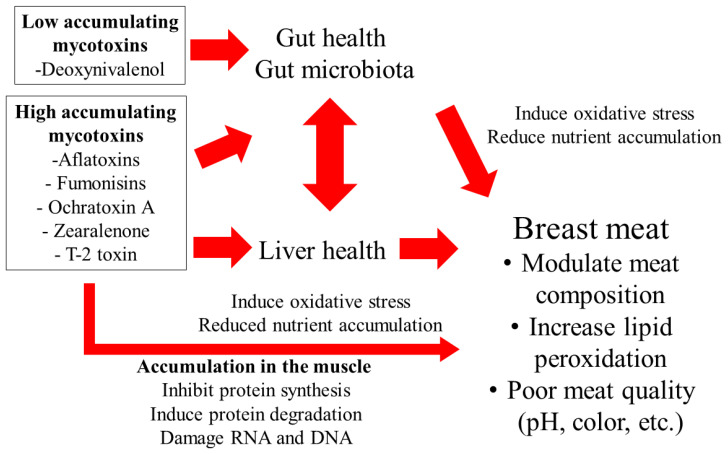
Proposed effects of low- and high- accumulating mycotoxins on the meat quality of broiler chickens. Low-accumulating mycotoxins negatively affect the gut health and gut microbiota of broiler chickens [96], and this can indirectly affect meat composition and quality by decreasing nutrient accumulation and inducing oxidative stress. In addition to indirect effects, such as low-accumulating mycotoxins, high-accumulating mycotoxins can negatively influence meat composition and quality by directly inhibiting protein metabolism, inducing protein degradation, and damaging RNA and DNA in the muscle [83,97].

Mycotoxins with high absorption and accumulation rates can remain in edible tissues (meat and liver). Hort et al. [93] showed that fumonisins were detected in the liver and fillet muscles. While zearalenone is known to have a higher absorption rate in the gastrointestinal tract of chickens [89], it was not detected in the liver or muscles, potentially due to rapid elimination after absorption [93]. However, Iqbal et al. [94] reported that 35, 41, and 52% of poultry meat samples were contaminated with aflatoxins, ochratoxin A, and zearalenone, respectively, in Pakistan. Mycotoxins in meat products can induce cancer and suppress the immune system in humans [95]. As shown in Figure 1, depending on the accumulating rate of mycotoxins, the effects of mycotoxins on meat quality can be different.

Table 5 summarizes the meat quality of broiler-fed feeds containing mycotoxins. Mycotoxins induce oxidative stress, which can lead to lipid peroxidation and negatively modulate the fatty acid composition and color of meat. However, results from Armanini et al. [98] may suggest that mycotoxins improve the meat quality of broiler chickens, potentially because growth retardation due to mycotoxins ameliorated the meat quality among fast-growing broilers [26].

### 2.6. Oxidized Oils and Fat

Oil and fat sources are important energy sources, and they are normally included, at levels of up to 5%, in broiler feed [103]. The freshness of oil and fat sources is important in poultry feed because oxidized oil and fat can induce lipid peroxidation and oxidative stress in animals [104]. Dong et al. [105] showed that the inclusion of oxidized oils induced oxidative stress and increased MDA in broiler chickens. Zhang et al. [106] demonstrated that broilers fed with oxidized oil had higher protein and lipid oxidation levels, increased WHC, and decreased pH in the breast meat post-mortem. Appropriate levels of fresh oil and fat sources should be included in broiler diets.

## 3. Effects of Nutritional Interventions on the Meat Quality of Broiler Chickens

### 3.1. Energy and Crude Protein

Recently, decreasing the energy levels and CP levels in poultry diets have drawn a lot of attention as options for reducing feed costs and improving energy and nutrient utilization in poultry production [107,108]. Energy and CP levels are key factors in determining the body composition of chickens [109]. The low- and high-energy levels of broiler diets can decrease and increase fat accumulation, respectively [110,111]. In contrast, a previous study by Lipiński et al. [112] reported that low-energy diets (−23.9 vs. −59.75 kcal/kg from the control feed) increased fat accumulation in the breast muscles, potentially as a defensive mechanism to cope with energy deficiency, while low energy diets reduced MDA concentrations in the breast meat. Nonetheless, Rosa et al. [113] reported that high-energy diets (2950 vs. 3200 vs. 3450 kcal/kg in the feed) induced greater fat accumulation in broiler chickens. Lipiński et al. [112] reported that low-energy diets increased WHC natural drip loss, pH, and lightness and reduced redness in the breast meat. In broilers fed low-energy diets, increased pH levels might be due to a lack of glycogen, which produces lactic acid and H^+^ ions post-mortem in the breast muscle [114]. Increased pH could lead to increased WHC in breast meat [115]. However, Arshad et al. [116] showed that low-energy diets (−100 kcal/kg in the starter feed and finisher feed) did not modulate pH, WHC, or lightness in the breast meat. 

According to Ciftci and Ceylan [117], the low CP levels (19.1% (normal) or 18.0% (low) for starter feed and 17.7% (normal) or 16.5% (low) for finisher feed) of the diets reduced the fat content of breast and thigh meat, potentially by reducing the energy available for fat accumulation. Brink et al. [118] reported that low CP diets (20.5% (high), 18.7% (medium), and 17.5% (low) in the grower phase and 19.5% (high), 18.0% (medium), and 16.6% (low) in the finisher phase feed) increased thawing loss, potentially by decreasing the myofiber density due to the bigger size of the myofiber. Consistently, Chodová et al. [119] demonstrated that low CP diets (21.6% (normal) and 20.39% (low) in the starter phase; 19.7% (normal) and 18.6% (low) in the grower phase; and 18.0% (normal) and 17.3% (low) in the finisher phase feed) increased drip loss in the breast meat. However, Park and Kim [120] showed that different CP levels (23% (normal) and 21% (low) in the starter phase and 20% (normal) and 18% (low) in the finisher phase) did not alter the pH, cooking loss, drip loss, or color of breast meat. The differences might originate from different animals, diets, experimental periods, etc.

### 3.2. Amino Acids (Aas)

Not only are AA important building blocks in the *de novo* synthesis of muscle proteins, but each AA also plays specific roles in modulating metabolic pathways, antioxidant systems, and enzymatic processes, which can potentially influence meat production and quality in animals [121]. Moreover, adding specific Aas above the recommended requirements became a common practice to exhibit beneficial effects in broiler chickens [122,123]. Methionine, the first limiting AA in corn–soybean meal diets for broilers, also plays an important role in improving the antioxidant system by being involved in the synthesis of glutathione (endogenous antioxidant) [124,125]. Moreover, Wen et al. [124] showed that the supplementation of methionine decreased the cooking loss and increased the pH in the breast meat, potentially by decreasing pyruvate kinase activity. Consistent with those findings, Albrecht et al. [126] reported that the supplementation of methionine (0.04, 0.12, and 0.32% in the feed) reduced cooking loss with increased pH levels in the breast meat and, in turn, a decrease in lightness. The supplementation of arginine has been known to reduce fat accumulation in chickens by altering de novo lipogenesis via regulating genes related to fat accumulation [20]. Our previous study showed that arginine at the requirement level improved lean muscle accumulation without affecting fat accumulation in broilers [127]. Arginine can be converted into nitric oxide, which can increase blood flow and can mitigate the incidence and severity of WS and SM abnormalities, potentially via reducing hypoxia in broiler chickens [128]. Moreover, nitric oxide has antioxidant effects, which have the potential to improve meat shelf life and attenuate lipid peroxidation in chicken meat [129]. Branched-chain amino acids (BCAAs), including valine, leucine, and isoleucine, play an important role in muscle protein synthesis because BCAAs are major essential AA in muscles [122,130,131]. Zhang et al. [132] demonstrated that the supplementation of BCAAs in low CP diets increased WHC, improved tenderness by reducing shear force, and increased intramuscular fat in finishing pigs. In chickens, Imanari et al. [133] showed that the supplementation of BCAA (isoleucine at 150% of the requirement and valine at 150% of the requirement) enhanced the free glutamine (e.g., the main taste-active component in meat) concentration in the breast meat of broiler chickens. A previous study by Kop-Bozbay et al. [134] showed that the dietary supplementation of BCAA ((1.0, 0.25, and 0.75 g/kg of L-leucine, L-isoleucine, and L-valine compared to the control (non-supplementation)) was detrimental for the meat yield of broilers under heat stress conditions, potentially due to antagonistic interactions between valine and isoleucine [134]. Zhang and Kim [135] reported that the supplementation of threonine (0.65, 0.70, 0.75, and 0.80 threonine to lysine ratios) did not influence meat quality in broiler chickens. While Aas are important building blocks and their specific functions have the potential to improve meat quality, the supplementation of Aas does not guarantee improvements in the meat yield or quality of broiler chickens.

### 3.3. Vitamins

The supplementation of vitamins is essential for broiler nutrition [68]. Whereas the supplementation of vitamin C is not required in broiler feed because the liver can synthesize enough, heat stress dramatically decreases vitamin C synthesis, and the supplementation of vitamin C can alleviate the negative effects of heat stress by fulfilling its requirement [136]. Vitamin E is an essential vitamin that should be supplemented in diets because it is not synthesized by chickens [137]. Both vitamins C and E are strong antioxidants in chickens and are known to alleviate oxidative stress induced by heat stress [24]. Gao et al. [138] and Pečjak et al. [139] reported that vitamin E supplementation (20 mg/kg feed (normal) vs. 200 mg/kg feed (high vitamin E)) improved antioxidative status and reduced lipid peroxidation in the breast meat of broiler chickens. The supplementation of vitamin E (10 mg/kg (normal) vs. 200 mg/kg (high vitamin E)) in the early-phase feed decreased the severity of WB and shear force in broiler chickens [140]. Mazur-Kuśnirek et al. [141] showed that the supplementation of vitamin E (200 mg/kg feed) decreased drip loss and enhanced WHC in broilers reared under high temperatures. However, Zeferino et al. [68] showed that the supplementation of vitamin C (257 mg/kg (normal) vs. 288 mg/kg (supplemented)) and vitamin E ((93 mg/kg (normal) vs. 109 mg/kg (supplemented)) did not influence the meat quality of broilers raised under heat stress conditions. Savaris et al. [142] reported that different levels of vitamin A (0, 6000, 16,000, 26,000, 36,000, and 46,000 IU/kg) and its supplementation period (D 0 to 21 vs. D 0 to 42) modulated the incidence and severity of WS in the breast meat and meat yield in broiler chickens, potentially via modulating cell metabolism in the muscle. Different forms of vitamin D, including 25-OHD3, 1,25(OH)2D3, and 1α(OH)D, modulated the color of the breast meat in broiler chickens [143]. Different rearing conditions can alter requirements (optimal doses), and different concentrations and forms of vitamins can affect the meat yield and quality of broiler chickens. 

### 3.4. Omega-3 Fatty Acid Sources

A number of studies have been conducted to increase the content of omega-3 fatty acids in chicken meat products [144]. Providing omega-3 fatty acid-rich sources in diets, including flaxseed oil, rapeseed oil, canola oil, fish oil, and marine algae, has been known to enhance the omega-3 fatty acid content in poultry meat products [145]. The supplementation of fish oil with conjugated linoleic acid at 2% in the feed reduced omega-6 fatty acids in the breast meat [146]. The supplementation of fish oil at 3% in the feed enhanced omega-3 fatty acids (docosahexaenoic and eicosatetraenoic acid) in the breast meat [147]. Moreover, Konieczka et al. [148] showed that the supplementation of fish, rapeseed, and flaxseed oils at 1% in the finisher phase increased the levels of omega-3 fatty acids in the breast meat. Moreover, Tompkins et al. [149] showed that material supplementation of fish oil at 2.3% in the feed enhanced the lean mass of offspring broiler chickens on D 42. Omega-3 fatty acids can improve blood flow in animals [150], which indicates that its supplementation can reduce hypoxia in fast-growing broilers and it has the potential to reduce the incidence and severity of WS and SM abnormalities. A previous study by Yang et al. [150] showed that dietary supplementation with fish oil at 2.5% in the feed instead of soybean oil reduced both fat accumulation and the ratio of omega-6 to omega-3 fatty acids and the ratio of polyunsaturated to saturated fatty acids, while it decreased lightness and increased drip loss in the breast meat of broilers. However, the supplementation of omega-3 fatty acid-rich oils in the feed is not cost-effective and can induce lipid peroxidation during storage [145]. Therefore, more studies are required to determine specific supplementation period and levels of omega-3 fatty acid sources in broiler productions. 

### 3.5. Probiotics and Prebiotics

Probiotics and prebiotics are defined as beneficial live microorganisms and non-digestible substances that can be food for beneficial bacteria, respectively [151,152]. The inclusion of probiotics and prebiotics in poultry feed became common to improve gut health and growth performance by enhancing gut microbiota in the poultry industry [153,154]. The supplementation of probiotics and prebiotics is known to reduce the incidence and severity of bacterial infection in the gastrointestinal tract of broiler chickens, which can improve meat safety [155,156,157]. Furthermore, the supplementation of *Bacillus cereus* var. toyoi (1 × 10^9^ CFU/g) and *B. subtilis* PB6 (2 × 10^7^ CFU/g) at 0.1% in the feed attenuated oxidative stress in broilers infected with *Salmonella* spp. [158]. Aluwong et al. [159] also showed that the oral administration of 1 mL of a yeast probiotic (*Saccharomyces cerevisiae*; 4.125 × 10^4^ CFU) improved the activities of glutathione peroxidase in the serum of broiler chickens. Improved antioxidant capacity has the potential to reduce lipid peroxidation in the breast meat of broiler chickens. Bai et al. [160] reported that the supplementation of *B. subtilis* fmbJ (4 × 10^10^ CFU/kg feed) improved endogenous antioxidant capacity and reduced MDA in the serum and liver, resulting in reduced drip loss and cooking loss in the breast meat. Probiotics may improve antioxidant capacity by reducing the colonization of pathogenic bacteria [161]. Mohammed et al. [162] reported that the supplementation of *B. subtilis* at 0.5 g/kg of feed reduced the cooking loss and pH and increased the taste of the leg meat of broiler chickens. The microbiota of the gastrointestinal tract of broiler chickens plays an important role in producing SCFA, which is an important energy source for the host [53]. Increased SCFA production levels, due to beneficially modulated microbiota, can affect the fat accumulation and body composition of broiler chickens [54]. However, a previous study by Wang et al. [163] showed that the supplementation of *Lactobacillus johnsonii* BS15 (1 × 10^6^ CFU/kg feed) reduced fat accumulation by downregulating fat accumulation-related genes (e.g., lipoprotein lipase) in adipose tissue and by upregulating hepatic genes related to fatty acid β-oxidation in broiler chickens. However, whether the mode of action of probiotics affects hepatic fat accumulation or oxidation is still unknown and demands more research.

### 3.6. Exogenous Enzymes 

There are diverse sorts of exogenous enzymes, including phytase, protease, carbohydrase, polysaccharidase, and lipase, and they have been supplemented in broiler feed to improve the growth performance, gut health, and nutrient utilization of broilers [164,165]. Exogenous enzymes have the potential to influence body composition by enhancing energy and protein utilization in broilers, but limited data are available thus far. Hajati [166] reported that the supplementation of enzyme cocktails (500 mg/kg of feed) containing arabinoxylanase, beta-glucanase, protease, and amylase increased the carcass yield and relative portion of thighs and drumsticks. Lu et al. [167] showed that the supplementation of protease (0.0125% of feed) reduced drip loss in the breast meat compared to the control group. Phytase, which became an essential feed additive for non-ruminant animals, hydrolyzes phytate-bound phosphorus (e.g., phytic acid; antinutritional factor) into free phosphate [168]. The supplementation of phytase can enhance energy and AA digestibility in broiler chickens [169]. However, many studies have reported that the supplementation of phytase did not influence the meat quality of broiler chickens [170,171,172]. The supplementation of exogenous enzymes may not significantly alter the meat quality or composition in broiler chickens.

### 3.7. Plant Polyphenolic Compounds (e.g., Plant Extracts and Essential Oils)

**Figure 2:**
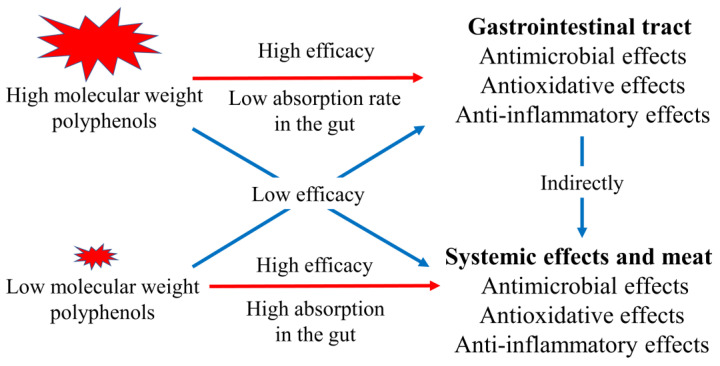
Proposed modes of action of polyphenols depending on their molecular weight and absorption rate in the gastrointestinal tract.

Plant extracts and essential oils, which contain diverse polyphenolic compounds (e.g., secondary plant metabolites), have obtained a lot of attention as alternative antibiotic growth-promoters in broiler production due to their antimicrobial, antioxidative, and anti-inflammatory effects [37,173,174]. Their antioxidative and anti-inflammatory effects have the potential to improve the meat quality of broiler chickens. Depending upon the molecular weight and absorption rate of polyphenols, their effects varied among broiler chickens, as shown in Figure 2.

Soldado et al. [175] suggested that high molecular weight polyphenolic compounds have the potential to exhibit systemic antioxidative effects by improving antioxidant status in the gastrointestinal tract and interacting with other antioxidants in animals. The beneficial effects of diverse polyphenols (e.g., plant extracts and essential oils) are shown in Table 6. The antioxidative effects of polyphenols would account for their beneficial effects on improving meat quality and decreasing lipid peroxidation in chicken meat [176]. However, some studies showed that the supplementation of polyphenol compounds did not influence the meat quality of broiler chickens [177,178]. Appropriate polyphenol sources (e.g., molecular weight) should be determined depending on the broilers’ raising conditions. 

Plant polyphenol compounds are known to affect fat accumulation in broiler chickens. Park and Kim [184] reported that the supplementation of *Achyranthes asper* (0.025% to 0.1% in the feed) reduced abdominal fat and increased breast meat portions in broiler chickens, and this could be due to its component, saponin, which might reduce lipid absorption in the gastrointestinal tract. Huang et al. [185] demonstrated that the oral administration (50 or 100 mg/kg of body weight) of green tea polyphenols decreased fat accumulation via downregulating genes related to fat accumulation and upregulating genes related to fat metabolism and transportation. Our previous study showed that the supplementation of tannic acid (0.5 to 2.5 g/kg of feed) reduced fat accumulation in broiler chickens by reducing the production of SCFA (e.g., microbial metabolites; important energy sources for the host) [54]. Therefore, the supplementation of polyphenolic compounds can positively affect the meat quality and body composition of broiler chickens. 

### 3.8. Organic Acids

Organic acids, considered to be alternatives for antibiotic growth promotors, are defined as organic compounds with acidic properties [186]. Most organic acids contain a carboxylic group (-COOH) including SCFA (≤C6), such as formic, acetic, propionic, fumaric, lactic, etc., but there are medium-chain fatty acids (C7 to C12) and long-chain fatty acids (>C12) [187]. Not only can organic acids improve food safety by showing antimicrobial effects [188] but animals also readily use organic acids as energy sources, which can affect the meat quality of broilers [189]. Galli et al. [190] showed that the supplementation of organic acids (blends of formic, phosphoric, lactic, acetic, butyric, and propionic acids; 0.75 to 3 g/kg of feed) reduced yellowness and improved the antioxidant status in the breast meat of broiler chickens. Ma et al. [191] showed that the supplementation of blends of formic acid, ammonium formate, acetic acid, propionic acid, and lactic acid (3 to 6 g/kg of feed) increased the meat pH and increased the ratio of polyunsaturated to saturated fatty acids in the breast meat. The supplementation of organic acids has the potential to alter the meat quality of broiler chickens.

## 4. Conclusions

Producing favorable-quality and lower-fat meat is important in broiler production to meet consumers’ desires and to be more competitive among other meat sources. However, a variety of different challenging conditions in poultry production have reduced meat yield and quality by reducing nutrient utilization and metabolism and inducing oxidative stress in broiler chickens. The supplementation of AA (e.g., arginine and BCAA) showed the potential to enhance the meat quality and body composition of broiler chickens by changing their metabolism. The supplementation of bioactive compounds, such as vitamins, probiotics, prebiotics, polyphenol compounds, and organic acids, improved the meat quality parameters, such as WHC, antioxidant capacity, and body composition, of broiler chickens. Potentially, plant polyphenolic compounds that have antioxidative, antimicrobial, and anti-inflammatory effects may be an effective nutritional strategy to improve meat quality and meat yield by improving nutrient utilization and reducing lipid peroxidation in broiler production. The development of nutritional strategies based on challenging conditions to meet market demands could play a key role in the improvement of the meat quality and composition of broiler chickens.

## Figures and Tables

**Table 1:** Differences in meat quality in slow- and fast-growing broilers.

Comparison Strains	Observations in Fast-Growing Broilers	Replicates per Treatments	References
Fast-growing (FG) vs. slow-growing (SG) chickens from La Gamme Hubbard (Sedar Co., Międzyrzec Podlaski, Poland)	A higher portion of breast and thigh yields Lower abdominal fats Higher fat and lower protein compositions Less yellowness	8	[29]
Medium slow-growing broilers (SG, Hubbard Red JA) vs. Ross308	Higher breast and lower thigh yields	10	[30]
Strains from S & G Poultry, Clanton, AL vs. Cobb-Vantress Inc., Siloam Spring, AR	Lower protein and higher fat contents Lighter, more redness, and less yellowness Higher pH Lower drip loss and higher thaw and cooking loss	80	[31]
Xueshan (Chinese local chickens) vs. Ross308	Lower pH Lighter and less redness and yellowness Lower protein and higher fat contents	6	[32]

**Table 2:** Effects of *Eimeria* infection on breast meat quality in broiler chickens.

*Eimeria* Species and Oocysts	Observations in the Breast Meat	Replicates per Treatments	References
1 × 10^4^ *Eimeria tenella*	Reduced redness Increased thiobarbituric acid reactive substances, a byproduct of lipid peroxidation	10	[58]
5 × 10^5^ mixture of *E. acervulina*, *E. maxima*, and *E. tenella*	Increased drip loss and cooking loss Decreased protein content Reduced redness Increased the formation of malonaldehyde, a final product of lipid peroxidation	10	[59]
4 × 10^4^ *Eimeria tenella*	Reduced lightness Reduced drip loss	5	[61]
4 × 10^4^ *Eimeria tenella*	Increased lightness	5	[60]

**Table 3:** Impact of heat stress on broiler meat quality.

Observations in the Breast Meat	Replicates per Treatments	References
Increased pH	10	[67]
Decreased cooking loss Increased thiobarbituric acid reactive substances, a byproduct of lipid peroxidation	12	[71]
Increased pH Increased lightness and decreased redness Increased cooking loss Decreased shear force	16	[68]
Increased pH Decreased lightness Increased yellowness Increased total lipid and reduced collagen Increased omega-3 polyunsaturated fatty acids	6	[65]
Reduced protein content and increased fat content Decreased pH Increased cooking loss Increased lightness and reduced redness Increased shear force	6	[66]

**Table 4:** Diverse kinds of mycotoxins, their absorption rates, and their effects on broiler chickens.

Mycotoxins	Absorption Rate in the Gastrointestinal Tract	Observations
Aflatoxins	Highly absorbable (80 to 90%) [74]	Induced mild negative effects on the gastrointestinal tract [75] Induced oxidative stress, damaged liver, damaged RNA and DNA, and suppressed immune system [76]
Deoxynivalenol	Low absorption rate [77]	Increased intestinal permeability [78] Damaged intestinal morphology and induced intestinal inflammation [79] Induced severe intestinal damage [80]
Fumonisins	Low absorption rate with quick plasma elimination but considerate accumulation rate [81,82]	Damaged ileum morphology and negatively affected gut microbiota [83] Induced mild inflammation in the gastrointestinal tract [84]
Ochratoxin A	High absorption rate [85]	Damaged RNA and DNA [86] Induced oxidative stress, lipid peroxidation, and cell apoptosis and inhibited protein synthesis [87] Suppressed immune system [88]
Zearalenone	High absorption rate [89]	Induced liver and kidney damage [90] Induced oxidative stress and suppressed immune system by damaging spleen [91,92]

**Table 5:** Effects of mycotoxins on the meat quality of broiler chickens.

Mycotoxins in the Feed	Observations in the Breast Meat	Replicates per Treatments	References
172 to 200 μg/kg ochratoxin A	Reduced pH Increased drip loss Reduced fat content Increased short-chain fatty acids and reduced polyunsaturated fatty acids Increased omega-6 to omega-3 fatty acid ratio	10	[87]
70 μg/kg aflatoxins B1	Increased yellowness	12	[99]
451 μg/kg aflatoxin B1, 684 μg/kg ochratoxin A, and 320 μg/kg of T-2 toxin	Decreased lightness Increased redness and yellowness	6	[100]
0.05 μg/kg aflatoxin and 20 μg/kg fumonisin	Increased lightness Reduced cooking loss Modulated fatty acid composition Stimulated antioxidant system as a defensive reaction	4	[98]
0.5 mg/kg aflatoxin	Increased thiobarbituric acid reactive substances, a byproduct of lipid peroxidation	15	[101]
43.2 mg/kg deoxynivalenol	Decreased lightness	8	[102]

**Table 6:** Effects of the supplementation of plant polyphenolic compounds (e.g., plant extracts and essential oils) on the meat quality of broiler chickens under different raising conditions.

Sources	Doses in the Feed	Bird Conditions/Replicates	Observations in the Breast Meat	References
Polyphenols originated from onions (quercetin and flavonols) and grape seeds (catechins, flavonols, pro-cyanidins, and anthocyanidins).	100 or 200 mg/kg	Heat-stressed/8	Reduced natural drip loss and water-holding capacity Increased crude ash Increased monounsaturated fatty acids and reduced omega-6 polyunsaturated fatty acids	[141]
		Fed rancid oils/10	Increased lightness Modulated fatty acid composition Increased crude fat and ash	[179]
		Fed diets contaminated with ochratoxin A/10	Enhanced water-holding capacity Modulated fatty acid composition (C12:0)	[87]
Bamboo leaf extract	1 to 5 g/kg	Normal condition/6	Improved antioxidant capacity and alleviated lipid peroxidation	[176]
Eucalyptus leaf polyphenol extract	500 mg/kg	Normal condition/4	Improved antioxidant capacity and increased redness	[180]
Cinnamaldehyde	50 mg/kg	Normal condition/8	Increased pH	[181]
A blend of essential oils: carvacrol, thymol, and cinnamaldehyde and curcumin	150 mg/kg	Normal condition/3	Improved antioxidant capacity and reduced thiobarbituric acid reactive substances, a byproduct of lipid peroxidation Reduced thawing loss Increased yellowness Reduced short-chain fatty acids and increased monounsaturated fatty acids and polyunsaturated fatty acids	[182]
Thymol and carvacrol	150 mg/kg	Normal condition/3	Reduced lipid peroxidation during storage	[183]

## Data Availability

No new data was generated in this manuscript.
